# Leveraging disease outbreak news to strengthen the global response to antimicrobial resistance: a call for action

**DOI:** 10.3389/fpubh.2025.1710596

**Published:** 2026-01-07

**Authors:** Reuben Kiggundu, J. P. Waswa, Herman Mwanja, Mackline Hope, Andrew Kambugu, Francis Kakooza, Dathan M. Byonanebye

**Affiliations:** 1 Infectious Diseases Institute, Makerere University, Kampala, Uganda; 2 Department of Community Health and Behavioural Sciences, School of Public Health, Makerere University, Kampala, Uganda

**Keywords:** antimicrobial resistance, disease outbreak news, outbreak, public health emergency, surveillance

## Abstract

Antimicrobial resistance (AMR) is an escalating global health threat, with low- and middle-income countries (LMICs) bearing the greatest burden as healthcare facilities become breeding grounds for resistant pathogens, leading to increased morbidity, mortality, and straining of already limited resources. The World Health Organization’s Disease Outbreak News (DONs) has proven invaluable for early warnings and coordinated responses to infectious disease outbreaks like Ebola and COVID-19, yet AMR events remain largely absent from this system, leading to under-detection, limited global visibility, and ineffective interventions. In this paper, we review the historical evolution of DONs, its supporting frameworks, and the dynamics of AMR outbreaks in LMIC healthcare settings to explore how DONs could be adapted for AMR. We recommend standardizing AMR outbreaks reporting, integrating DONs into response efforts, linking AMR surveillance to DONs workflows, and expanding the definition of Public Health Emergencies of International Concern (PHEIC) to include high-morbidity AMR events, steps that would elevate AMR from a “silent pandemic” to a visible priority.

## 1 Introduction

Antimicrobial resistance (AMR) is a growing global health crisis that threatens the foundations of modern medicine (1). The global burden of deaths, disease, and economic loss attributable to AMR continues to rise, with low- and middle-income countries (LMICs), where health systems are under-resourced and surveillance capacities limited, bearing the greatest impact (2). AMR outbreaks, as defined by the World Health Organization (WHO), are characterized by sudden increase in infections caused by antimicrobial-resistant organisms that exceeds the expected baseline within a given setting, requiring urgent detection, investigation, and control measures (3). Yet unlike traditional infectious disease outbreaks such as Ebola, where rapid identification and response systems are well established, most health systems in LMICs remain insufficiently equipped to effectively detect, contain, and manage AMR outbreaks. Healthcare facilities (HCFs) in LMICs are critical hotspots for AMR outbreaks, with multidrug-resistant organisms increasingly reported, resulting in prolonged hospital stays, excess mortality, and significant financial strain on already constrained health systems (4, 5). While platforms such as ProMED-AMR contribute to disseminating AMR-related outbreak information across human, animal, and environmental domains, they are not solely dedicated to reporting AMR outbreaks and therefore lacks the focused mandate, visibility, reach, and institutional authority of the WHO’s Disease Outbreak News (DONs) (6, 7). Consequently, AMR outbreaks remain under-detected, under-reported, and insufficiently disseminated globally compared with traditional infectious disease epidemics, highlighting the urgent need for a dedicated platform (8).

The WHO DONs provide information on confirmed acute public health events or potential threats of concern (9). The DONs are the official WHO public reporting system for global disease outbreaks. Established in 1996, it compiles event-based information from countries and other partners, which WHO organizes into narrative reports detailing outbreak circumstances. These reports outline the affected geographic region, identify a known or suspected disease (or an unexplained syndromic event) after it has been made public by the respective country, and provide insights into response measures, such as laboratory testing (9). The DONs are used by WHO to fulfill requirements on information sharing about disease outbreaks as required by article 11.4 of the International Health Regulations (2005; IHR) (9, 10). The DONs have played a crucial role in raising awareness about emerging and re-emerging infectious diseases, serving as an early warning system for outbreaks that require immediate attention and, in the process, built capacity for global health security (8). The initial days of an outbreak are critical in preventing its escalation into an epidemic or pandemic (11). Early warnings enable countries to access resource stockpiles, secure funding, utilize decision support tools, coordinate cross-border surveillance and response efforts, and prepare the public for potential threats and response actions (12, 13). By disseminating timely, authoritative, and independent information, DONs have contributed to the global response to infectious disease threats such as Ebola, COVID-19, and cholera (9). Given their success in alerting public health authorities and mobilizing resources for outbreak control, the integration of DONs into AMR surveillance and response plays a critical role. Despite the high burden and mortality associated with AMR, dedicated AMR DONs remain few and far apart (14–16), leading to its characterization as a silent pandemic (17). AMR-related infections continue to rise globally, threatening the effectiveness of existing antibiotics and increasing morbidity and mortality rates, yet the urgency to address this crisis is still lacking (18). Adopting a DONs-like approach for AMR outbreaks in HCFs could help bridge this gap by ensuring that AMR outbreaks are identified, reported, and acted upon in a timely manner.

Hospitals are critical points for detecting and responding to AMR outbreaks, as they are often the first to identify resistant infections. Implementing a DONs system would ensure that whenever there is an AMR outbreak in HCFs, hospital administrators and healthcare workers generate a local disease outbreak news report. This report would document the resistant pathogen involved, the affected patient population, and potential sources of transmission. This report could then be shared with the Ministry of Health to support any response efforts. By systematically reporting AMR outbreaks in the same way that infectious disease outbreaks are documented, hospitals could enhance their ability to implement rapid infection prevention and control (IPC) measures, including antimicrobial stewardship (AMS) interventions (19).

As with other infectious disease outbreaks, AMR outbreaks require immediate containment strategies, including enhanced diagnostic capacity, optimized antimicrobial prescribing practices, and stricter infection control measures (20). Furthermore, integrating DONs into AMR surveillance would help drive public and institutional awareness, fostering a sense of urgency in combating resistant infections. The regular publication of AMR DONs would also reinforce the need for action at multiple levels, from hospitals implementing local interventions to policymakers enacting stronger regulations on antibiotic use in healthcare and agriculture. In this paper, we describe how DONs can enhance the detection, response, and control of AMR outbreaks in HCFs and advocate for their integration into AMR outbreak management.

## 2 Evolution of disease outbreak news

**Figure 1:**
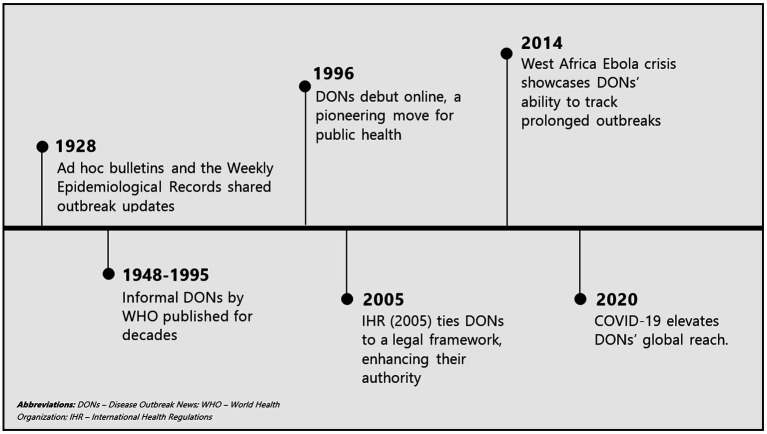
Key milestones in the origins and evolution of DONs.

Key milestones in the origins and Evolution of DONs are shown in Figure 1. Early efforts for reporting disease outbreaks initiated in 1926 relied on *ad hoc* bulletins and the Weekly Epidemiological Record (WER) to track cholera, plague, smallpox, typhus, and yellow fever (21). This fragmented coordination system was slow and lacked standardization, reflecting the limits of pre-digital communication (22). Efforts toward centralized reporting began informally through traditional methods like mail and fax, driven by the need for timely, centralized global alerts (22, 23) began following establishment of the WHO in 1948. A significant evolution occurred in 1996, with WHO pioneering online DONs publications, leveraging internet technology to revolutionize public health communication (22). This shift was driven by technological advancements (internet, information technology infrastructure); globalization trends (increased international travel and trade); and reforms following the 1995 Ebola outbreak in Zaire which exposed gaps in global notification and prompted formalization of reporting (24). These first DONs were simple text-based alerts, often linked to WER summaries and focusing only on acute epidemics with pandemic potential.

The 2005 revision of the IHR, following the 2003 SARS outbreak (25), the role of DONs was significantly enhanced by legally mandating WHO member states to notify WHO about public health emergencies of international concern (PHEICs) within 24 hours of detection (22). This binding legal framework gave DONs increased global authority and urgency. Subsequently, DONs became synonymous with high-profile global outbreaks like H5N1 avian influenza (2005) (26) and the H1N1 pandemic (2009) (27) reflecting emphasis on zoonotic and emerging viral threats. In the 2010s, prolonged outbreaks such as the West African Ebola epidemic (2014–2016) (28), Zika virus epidemic (2015–2016) (29), and Middle East Respiratory Syndrome (index outbreak in 2012) (30) underscored the ability of DONs in tracking prolonged outbreaks. During this period, the standardized DONs structure, comprising event description, risk assessment, and response actions emerged, supported by WHO’s Global Outbreak Alert and Response Network (GOARN) (31).

The 2020 COVID-19 pandemic represented a pinnacle of DONs visibility, beginning with the initial notification in January 2020 and followed by frequent updates accessed by millions globally (32). Throughout the early 2020s, DONs expanded coverage to complex public health events such as Mpox (2024) (33), yet the lack of coverage for AMR outbreaks persisted. As of 2025, DONs remain a cornerstone of WHO’s epidemic intelligence, disseminated digitally alongside comprehensive risk assessments (34). Evolving from basic notices to structured, authoritative outbreak communications, DONs continue to primarily address acute PHEICs without significant focus on AMR outbreaks. Over decades, DONs have transitioned from niche bulletins into indispensable tools in global health security, continuously adapting to emerging pathogens, technological advancements, and shifting public health needs, however, they have yet to fully incorporate AMR outbreaks in their evolution.

## 3 Supporting frameworks for DONs

The creation and dissemination of DONs rely on interconnected frameworks embedded within global health security and surveillance systems. Central to this is the IHR (2005), a binding agreement among at least 190 countries that mandates timely collaboration in detecting, preventing, and managing public health threats transcending national borders (35). Countries are obligated to notify WHO within 24 h about any PHEICs, and DONs are a key mechanism for WHO to fulfill this communication obligation (22). Supporting the generation of accurate and timely DONs is the WHO’s GOARN, a network comprising institutions and experts collaborating on outbreak detection, verification, and response, thus ensuring robust technical inputs and validated data (31). Additionally, Event-based surveillance (EBS), which involves systematic monitoring of informal sources such as news reports, social media, and community rumors, often provides initial alerts to potential health events, prompting further investigation (12). Conversely, Indicator-based surveillance (IBS), involving structured and verified health data analysis from health systems (e.g., healthcare facilities), contributes reliable and confirmed information, serving as a backbone for DONs (36).

The WHO’s Epidemic Intelligence Framework systematically guides early outbreak detection, emphasizing speed and accuracy in data collection, verification, analysis, risk assessment and communication (37). The framework enables rapid and informed response decisions, integrating input from both EBS and IBS. Given that numerous outbreaks involve multiple sectors, especially animal-human transmission (zoonotic diseases like Ebola and avian influenza) or environmental contamination (e.g., cholera), integrating One Health approaches within DONs has facilitated comprehensive and multi-sectoral containment strategies (38). Robust national public health systems, including effective Public Health Emergency Operations Centers (EOCs) (39), are crucial to DONs efficacy. The reliability and timeliness of data submitted from national authorities directly influence DONs accuracy and effectiveness, highlighting the importance of strengthening national EOCs. Additional frameworks that guide DONs include WHO Emergency Response Framework (ERF) (40), Global Health Security Agenda (GHSA) (41), Integrated Disease Surveillance and Response (IDSR) strategy (42), and Early Warning and Response Network (EWARN) (43). Although, PHEIC lists have historically excluded AMR outbreaks (44), establishing mandatory reporting of drug-resistant infections, from bottom level (HCFs) up to national authorities and ultimately to WHO, could strengthen surveillance capacity, generate interest and public awareness, and foster a shift in perception of AMR from a “silent pandemic” pandemic to a visible and urgent global health threat.

## 4 Disease outbreak news for AMR outbreaks

### 4.1 AMR outbreaks in healthcare facilities

An AMR outbreak in a HCF is characterized by an unexpected increase in infections caused by antimicrobial-resistant pathogens within a specific timeframe and location (45). Epidemiologically, these outbreaks are driven by factors that enhance infection transmission between patients, health care workers, selective pressure from antibiotic use, and introduction from the community or other facilities (46). Diagnostic confirmation involves clinical suspicion, complemented by laboratory antimicrobial susceptibility testing (AST), polymerase chain reaction (PCR) for resistance genes, or whole-genome sequencing to determine epidemiological linkages (47). AMR outbreaks lead to increased morbidity, mortality, prolonged hospital stays, and higher healthcare costs, particularly when involving pathogens on the WHO’s Bacterial Priority Pathogens List (45, 48).

AMR outbreaks in HCFs manifest as unexpected clusters of infections or colonization caused by the same resistant pathogen. These outbreaks commonly implicate multidrug-resistant organisms like methicillin-resistant *Staphylococcus aureus* (MRSA) (49), vancomycin-resistant Enterococcus (VRE) (50), carbapenemase-producing Enterobacterales (CPE) such as *Klebsiella pneumoniae* (51), and multidrug-resistant Gram-negative bacteria including *Pseudomonas aeruginosa* and *Acinetobacter baumannii* (52), which can spread via healthcare worker hands, contaminated equipment, or environmental surfaces. Effective detection of AMR outbreaks depends on robust passive surveillance through routine microbiology reports and clinical alerts to identify unusual patterns, complemented by active measures such as healthcare-associated infection (HAI) surveillance and environmental sampling (53). This requires strong laboratory capacity for phenotypic identification, AST, and rapid assays such as *CarbaNP* for carbapenemases (54), alongside advanced tools like whole-genome sequencing to trace transmission chains and distinguish outbreak strains (55), however, such capacities are not universally available in LMICs, limiting the effectiveness of AMR surveillance and outbreak detection (56). Equally critical are dedicated HCF systems, including AMS programs that optimize antibiotic use to limit resistance selection, and IPC programs that implement measures such as hand hygiene, environmental cleaning, transmission-based precautions, and multimodal audits to prevent further spread (57, 58).

The true prevalence of AMR outbreaks in healthcare settings remains poorly documented, probably due to inadequate systematic surveillance systems in HCFs, particularly in LMICs, and absence of requirements for DONs to report AMR outbreaks (56). Recommended AMR outbreak management involves structured processes, involving data collection, analysis, interpretation, and coordinated communication among outbreak-management teams, leadership of healthcare facilities, and healthcare staff (3). However, these recommendations do not explicitly incorporate DONs as part of the communication strategy. Highlighting this oversight is the WHO-issued DONs regarding Carbapenem-resistant *Pseudomonas aeruginosa* infections in Mexico, triggered by rising infection rates among American patients who had undergone surgery in Mexico (medical tourism). Interestingly, similar infections occurring among local Mexican patients in the same facilities were overlooked, illustrating disparities in outbreak reporting (59). These underscore both the transboundary risk posed by AMR and the critical need for inclusive and comprehensive DONs reporting for AMR outbreaks to enhance global awareness, equity, and effective containment measures.

### 4.2 Alert thresholds for reporting AMR outbreaks in healthcare facilities

Establishing clear thresholds for reporting AMR outbreaks in HCFs remains critical to balancing timely detection with practical feasibility. Globally, guidelines often prioritize deviations from baseline resistance rates or novel resistance mechanisms. For instance, the WHO recommends triggering alerts when AMR infection rates exceed historical averages by 2 to 3 standard deviations or when pathogens with new resistance genes emerge unexpectedly (3). Similarly, the European Center for Disease Prevention and Control (ECDC) mandates immediate reporting for single cases of pan-resistant pathogens like carbapenem-resistant *Acinetobacter baumannii* in regions where they are not endemic (10). However, challenges persist in low-resource settings where inconsistent surveillance data make baseline thresholds difficult to define (56), leading some hospitals to adopt WHO generic guidelines (45) that may not reflect local epidemiology. Pathogen-specific risks also influence thresholds: high-priority threats such as MRSA outbreaks might focus on clusters (such as ≥3 linked cases in a week) rather than isolated incidents (3, 53). Integrating these tiered thresholds into DONs frameworks in each given local context could standardize AMR reporting, ensuring outbreaks are flagged early without overwhelming systems, a key step toward bridging the gap between local detection and global response.

### 4.3 Current role of DONs in AMR outbreaks

The primary focus of DONs is the rapid dissemination of information about confirmed outbreaks requiring immediate action to curb their spread and impact. However, AMR is seldom included in DONs despite considerable impact of AMR outbreaks in HCFs, farms, or communities, with dedicated DONs remaining scarce and inconsistent (14–16). This reflects DONs’ design, which prioritizes outbreaks meeting the threshold for PHEICs (60); however, AMR outbreaks are often perceived as slow, chronic, and localized (61, 62), and thus fail to meet this threshold.

Many LMICs frequently experience significant disease outbreaks compounding both AMR and epidemic burdens (63). The widespread and often inappropriate use of antimicrobials during disease outbreaks (64, 65), driven by diagnostic limitations, weak regulatory systems, and concerns about secondary infections (66), accelerates the emergence of resistant pathogens (67), highlighting the consequences of neglecting AMR in outbreak responses and reporting. Furthermore, AMR interacts closely with and is enhanced by emerging global threats, including climate change (Magnano San (68)), social inequalities (69), armed conflicts (70), humanitarian crises (71), and microplastic pollution (72), all of which further exacerbate disease outbreaks (73). In turn, AMR undermines effective outbreak response, adversely impacting outcomes and impact. Like other PHEICs, AMR respects no borders and is amplified by globalization (41), necessitating coordinated global response strategies such as integration into DONs. Despite these parallels, AMR outbreaks remain inadequately reported, delaying essential interventions.

The WHO recommends continuous communication and information sharing among healthcare workers and outbreak management teams as essential for effectively coordinating responses, enabling timely interventions, and enhancing awareness during AMR outbreaks in HCFs (3). However, this guidance does not explicitly include DONs, a critical WHO tool for outbreak communication, thereby overlooking their potential utility in hospital-based AMR outbreak management. This omission undermines the potential benefits of DONs, such as working as an early warning system, amplifying risk communication, and raising public awareness and response preparedness, and mobilizing resources functions that could effectively link local containment efforts with broader response mechanisms (national and international), particularly for outbreaks involving WHO priority bacterial pathogens (45). Excluding AMR outbreaks in DONs significantly limits local and international awareness during an era characterized by intense globalization, undermining efforts for coordinated responses, and potentially enabling unchecked outbreak transmission. This was exemplified by the international spread of *NDM-1 (New Delhi metallo-beta-lactamase-1)*-producing bacteria, initially identified in India in 2008 (74), and the emergence of the *mcr-1* gene conferring colistin resistance in *Enterobacteriaceae* species, first detected in China in 2015 (75). Despite their serious global health implications, neither event triggered timely DONs. Without creating systems that amplify the impact of and action to combat AMR within health facilities, AMR will remain a “silent” pandemic.

The recent global alert on hypervirulent carbapenem-resistant *Klebsiella pneumoniae* emphasized both AMR and surveillance needs (76). Notably, the DONs were initiated in response to a high-level information request from the Global Antimicrobial Resistance and Use Surveillance System (GLASS) on Emerging Antimicrobial Resistance Reporting (GLASS-EAR) to national WHO GLASS focal points. This highlights an important gap: the alert was reactive to a specific global request rather than proactively initiated by HCFs or national surveillance systems. Furthermore, the DONs provided a broad global overview of prevalence rather than explicitly announcing a localized epidemic or pandemic event, emphasizing persistent limitations in current DONs frameworks. However, we believe that AMR DONs should be initiated at the health facility level.

AMR outbreaks in hospitals are documented in scientific literature and reports, yet these rarely appear as standalone DONs. For example, a 2022 outbreak of multi-drug-resistant *Acinetobacter baumannii* in a European hospital did not trigger DONs (77), despite its severity and cross-border implications (10). Similarly, the rise of extensively drug-resistant Shigella in the United States has not prompted any DONs, possibly because it is seen as a chronic trend rather than a sudden outbreak (78). However, the recent (2025) Queensland melioidosis outbreak tied to flooding and soil bacteria *(Burkholderia pseudomallei)* made it into DONs, but its AMR aspect was not emphasized (79), despite known resistance challenges (80). Similarly, during the 2011 cholera outbreak in Haiti and the Dominican Republic, resistant organism strains were documented by researchers, yet DONs did not prioritize the resistance aspects of the outbreak (81). The WHO GLASS treats AMR as a cross-cutting issue distinct from traditional disease outbreak surveillance frameworks and associated DONs systems (82). This distinction implies that DONs are not currently designed as the primary vehicle for AMR-specific alerts. A 2021 review of IHR implementation by WHO further highlighted this gap, noting that AMR remains underreported as an independent public health threat, advocating instead for its integration into broader surveillance systems, not AMR-specific DONs (83). This suggests a pattern: DONs focus on the pathogen and acute event, not the resistance profile, unless it is critical to the outbreak narrative or control measures. We believe a system mirroring DONs, initiated at the HCF level, will play a critical role in efforts to create awareness about AMR and support the global AMR response efforts.

### 4.4 Untapped potential: leveraging DONs for AMR outbreak management

**Figure 2:**
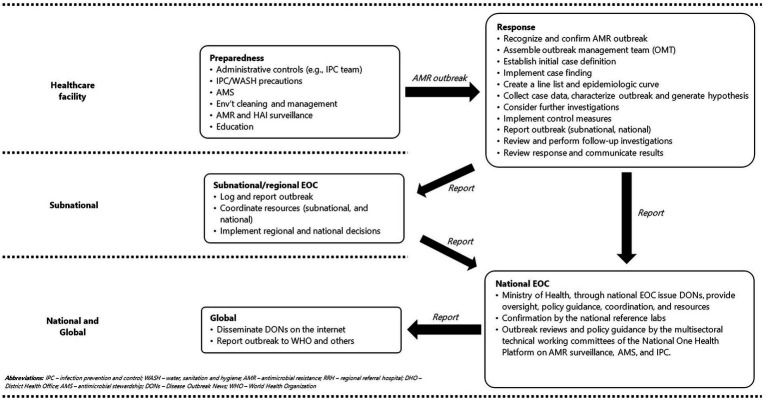
Proposed workflow for generating DONs and reporting to the national level following an AMR outbreak in Ugandan healthcare facilities.

DONs can play a crucial role in managing AMR outbreaks in HCFs, significantly enhancing both local (at the health facility level), national, and international responses, as shown in Figure 2. The HCFs facilities should maintain robust systems for monitoring HAIs and AMR in anticipation of potential AMR outbreaks. Once an outbreak is detected, the HCF initiates an immediate response following WHO guidance (3). The outbreak is reported to the subnational (regional EOC) and subsequently to national EOC through established public health emergency reporting systems (84). The decentralized EOCs and the national EOCs institute response mechanisms, providing policy guidance, mobilizing resources, and ensuring technical support. National microbiology reference laboratories play a critical role in confirming the outbreak, identifying genetic resistance mechanisms, and offering organism-specific containment recommendations. The MOH and multisectoral technical working committees of the National One Health Platform, such as those for AMR surveillance, AMS, and infection prevention and control, conduct reviews to ensure coordinated, evidence-based responses and provide comprehensive policy guidance aligned with One Health principles.

As an early warning tool, DONs can rapidly alert both the local and global community to new and emerging AMR threats with potential for cross-border spread, enabling timely response actions. They can stimulate coordinated responses by mobilizing resources, technical expertise, and support through networks like the GOARN, which is particularly beneficial for resource-limited settings that might otherwise struggle to manage AMR outbreaks effectively. Additionally, DONs can enhance risk communication by providing clear, authoritative information to healthcare providers and the public on the severity of AMR outbreaks and recommended preventive practices such as hand hygiene to limit spread. Beyond their immediate impact, DONs also offer a strategic platform to promote the One Health approach. By highlighting AMR outbreaks, DONs can emphasize the interconnectedness of human, animal, and environmental health, informing comprehensive approaches and multisectoral collaboration. Furthermore, DONs can enable early tracking of emerging AMR threats by spotlighting novel resistance mechanisms, such as new plasmids identified in AMR outbreaks, thus informing early-stage research and facilitating the timely development of countermeasures. To optimize the utility of AMR DONs, it is imperative to integrate practical elements such as real-time AMR data analysis, simplified dashboards, and integration into current health management information systems for reporting.

## 5 Recommendations and future direction

To effectively harness DONs’ utility for managing AMR outbreaks, several practical approaches to adapt the WHO DONs framework are essential. Successful implementation of any AMR outbreak response, including reporting, investigation, containment, and notification, depends fundamentally on the ability to detect the outbreak through timely and reliable microbiological confirmation. Strengthening microbiology laboratory capacity within healthcare facilities and national laboratory networks is therefore the cornerstone for effective AMR outbreak detection and response. This laboratory strengthening should be integrated with simultaneous reinforcement of AMS and IPC programs at the facility level, establishing robust healthcare facility–based systems that ensure effective oversight, coordination, and rapid decision-making for all AMR outbreak response actions. These programs must be guided by standardized national or locally endorsed guidelines that are tailored to local epidemiology and resource availability, and regularly updated using facility-specific antibiograms and surveillance data to ensure contextually appropriate, evidence-based interventions. Furthermore, HAI surveillance should be integrated into facility and national IPC and AMS programs as a core component of AMR surveillance, enabling early detection of unusual clusters of resistant pathogens, providing real-time feedback on the effectiveness of AMS and IPC measures, and facilitating prompt recognition and response to AMR outbreaks before widespread transmission occurs.

Secondly, there is a need for the Ministries of Health to roll out a reporting system for AMR outbreaks at the health facility level. This system should then be linked to the national level and potentially to the international level if reporting criteria are met. Secondly, there is a need to define and standardize international reporting requirements (including thresholds) for reporting AMR outbreaks at all levels (health facility, national, and international). Additionally, the definition of a PHEIC within the IHR framework should be expanded to encompass AMR events characterized by high morbidity or resistance to last-line drugs, even if they spread more slowly than traditional disease outbreaks. Integrating data streams is another critical approach, as linking AMR DONs more closely with the GLASS system and hospital-based surveillance systems could enable earlier detection of AMR outbreaks. Furthermore, lowering the threshold for issuing DONs to include AMR events in special circumstances that enhance transmission, such as regions (such as crowded spaces like cities and refugee settlements), special populations (such as immunocompromised), and association with traditional epidemics and pandemics.

However, implementing these changes comes with challenges that must be anticipated and mitigated. The gradual nature of AMR might lead to perceptions that frequent DONs are alarmist, potentially diluting their authority and impact if not carefully calibrated. Capacity constraints also pose a crucial challenge, as many countries, particularly LMICs, lack the real-time detection capabilities needed to supply timely data for DONs, which could delay alerts. Significant cost challenges also exist, including the financial burden of strengthening surveillance systems, training personnel, and maintaining the infrastructure required for continuous monitoring and timely dissemination of AMR DONs. Moreover, there is a risk associated with scope expansion; if DONs shift too far from their traditional focus on acute epidemics to encompass slow issues like AMR, they might lose their acute impact.

## 6 Conclusion

Disease Outbreak News holds untapped potential to transform the management of AMR outbreaks in healthcare facilities, bridging local challenges with global responses. AMR, similar to traditional epidemics and pandemics, transcends national borders, a phenomenon becoming increasingly significant in today’s highly globalized world. While traditionally focused on acute, internationally significant outbreaks like Ebola or COVID-19, the existing WHO DONs framework can be adapted to address the rising threat posed by AMR outbreaks by serving as an early warning system, enhancing collaborative responses, amplifying risk communication, integrating One Health perspectives, and tracking emerging resistance threats. However, realizing this potential requires practical adaptations such as expanding outbreak definitions, integrating data streams, standardizing AMR reporting, and lowering regional thresholds while overcoming challenges like perception, capacity gaps, and scope expansion. By incorporating these changes, particularly in LMICs where surveillance is weak, DONs could elevate local and global awareness and response actions against AMR outbreaks, reducing their impact.

## References

[1] Prestinaci F Pezzotti P Pantosti A. Antimicrobial resistance: a global multifaceted phenomenon. Pathogens Global Health. (2015) 109:309–18. doi: 10.1179/2047773215Y.0000000030, 26343252 PMC4768623

[2] Naghavi M Vollset SE Ikuta KS Swetschinski LR Gray AP Wool EE . Global burden of bacterial antimicrobial resistance 1990–2021: a systematic analysis with forecasts to 2050. Lancet. (2024) 404:1199–226. doi: 10.1016/S0140-6736(24)01867-1, 39299261 PMC11718157

[3] World Health Organization (WHO). Responding to outbreaks of antimicrobial-resistant pathogens in health-care facilities: guidance for the Western Pacific region; Manila. Manila: World Health Organization Regional Office for the Western Pacific (2022).

[4] Founou RC Founou LL Essack SY. Clinical and economic impact of antibiotic resistance in developing countries: a systematic review and Meta-analysis. PLoS One. (2017) 12:621. doi: 10.1371/journal.pone.0189621, 29267306 PMC5739407

[5] Pace MC Corrente A Passavanti MB Sansone P Petrou S Leone S . Burden of severe infections due to Carbapenem-resistant pathogens in intensive care unit. World J Clin Cases. (2023) 11:2874–89. doi: 10.12998/wjcc.v11.i13.2874, 37215420 PMC10198073

[6] Bowman-Derrick S. Introducing ProMED-AMR. Int J Infect Dis. (2022) 116:S1. doi: 10.1016/j.ijid.2021.12.002

[7] Sirdar MM Hanson J Maxwell J Matar GM Myaing TT Holmes A. Antimicrobial resistance (AMR): ISID’S global efforts to bring light to the silent pandemic. IJID One Health. (2024) 5:491. doi: 10.1016/j.ijregi.2024.100491 PMC11652920 39698050

[8] Carlson CJ Boyce MR Dunne M Graeden E Lin J Abdellatif YO . The World Health Organization’s disease outbreak news: a retrospective database. PLOS Global Public Health. (2023) 3:e0001083. doi: 10.1371/journal.pgph.0001083, 36962988 PMC10021193

[9] World Health Organization (WHO). (2023). “Disease outbreak news (DONs).” Emergencies. 2023. Available online at: https://www.who.int/emergencies/disease-outbreak-news (accessed April 1, 2025).

[10] European Centre for Disease Prevention and Control (ECDC). Carbapenem-resistant *Acinetobacter Baumannii* in healthcare settings – 8 December 2016. Stockholm: ECDC (2016).

[11] National Academies of Sciences, Engineering and Medicine. (2016). “Global health risk framework: Resilient and sustainable health systems to respond to global infectious disease outbreaks: Workshop summary.” Available online at: https://www.ncbi.nlm.nih.gov/books/NBK367950/?utm_source=chatgpt.com (accessed April 22, 2025). 27308691

[12] McKnight CJ Aboushady AT Lane CR. Beyond early warning: towards greater granularity in the use of event-based surveillance for public health emergencies. BMC Public Health. (2024) 24:3488. doi: 10.1186/s12889-024-20963-2, 39696107 PMC11656584

[13] Morse SS Mazet JAK Woolhouse M Parrish CR Carroll D Karesh WB . Prediction and prevention of the next pandemic zoonosis. Lancet. (2012) 380:1956–65. doi: 10.1016/S0140-6736(12)61684-5, 23200504 PMC3712877

[14] World Health Organization (WHO) In: WHO, editor. Disease outbreak news | 1997 - vancomycin resistant *Staphylococcus Aureus*. Geneva: (1997)

[15] World Health Organization (WHO) In: WHO, editor. Disease outbreak news | extensively drug-resistant tuberculosis (XDR-TB) in United States of America air passenger. Geneva: (2007)

[16] World Health Organization (WHO). Disease outbreak news | extensively drug-resistant *Shigella Sonnei* infections - Europe - European region (EURO). Geneva: WHO (2022).

[17] Laxminarayan R Matsoso P Pant S Brower C Røttingen JA Klugman K . Access to effective antimicrobials: a worldwide challenge. Lancet. (2016) 387:168–75. doi: 10.1016/S0140-6736(15)00474-2, 26603918

[18] Murray CJL Ikuta KS Sharara F Swetschinski L Robles Aguilar G Gray A . Global burden of bacterial antimicrobial resistance in 2019: a systematic analysis. Lancet. (2022) 399:629–55. doi: 10.1016/S0140-6736(21)02724-0, 35065702 PMC8841637

[19] Goff DA Kullar R Goldstein EJC Gilchrist M Nathwani D Cheng AC . A global call from five countries to collaborate in antibiotic stewardship: united we succeed, divided we might fail. Lancet Infect Dis. (2017) 17:e56–63. doi: 10.1016/S1473-3099(16)30386-3, 27866945

[20] Arthur RR LeDuc JW Hughes JM. Global surveillance for emerging infectious diseases In: Richard L Guerrant David H Walker Peter F Weller. Tropical infectious diseases: Principles, pathogens and practice. Philadelphia, PA: Saunders Elsevier (2011). 105–9.

[21] World Health Organization (WHO). Weekly epidemiological record, 1929, vol. 04, 20 [full issue]. Weekly Epidemiological Record = Relevé épidémiologique Hebdomadaire. (1929) 4:215–24.

[22] Snowden FM. Emerging and reemerging diseases: a historical perspective. Immunol Rev. (2008) 225:9–26. doi: 10.1111/j.1600-065X.2008.00677.x, 18837773 PMC7165909

[23] Dato V Shephard R Wagner MM. Outbreaks and investigations In: Michael M Wagner Andrew W Moore Ron M Aryel. Handbook of biosurveillance, Cambridge, MA: Academic Press vol. 13 (2007)

[24] Bell BP Damon IK Jernigan DB Kenyon TA Nichol ST O’Connor JP . Overview, control strategies, and lessons learned in the CDC response to the 2014-2016 Ebola epidemic. MMWR Suppl. (2016) 65:503. doi: 10.15585/mmwr.su6503a2 27389903

[25] Cherry JD Krogstad P. SARS: the first pandemic of the 21st century. Pediatr Res. (2004) 56:1–5. doi: 10.1203/01.PDR.0000129184.87042.FC, 15152053 PMC7086556

[26] World Health Organization (WHO) In: WHO, editor. Disease outbreak news | 2005 - Viet Nam. Geneva: (2005)

[27] World Health Organization (WHO) In: WHO, editor. Disease outbreak news | 2009 - Ireland. Geneva: (2009)

[28] Kyobe Bosa H Kamara N Aragaw M Wayengera M Talisuna A Bangura J . The West Africa Ebola virus disease outbreak: 10 years on. Lancet Glob Health. (2024) 12:129. doi: 10.1016/S2214-109X(24)00129-3, 38527467

[29] World Health Organization (WHO) In: WHO, editor. Zika Virus Disease Outbreak 2015–2016. Geneva: (2015)

[30] World Health Organization (WHO) In: WHO, editor. Middle East respiratory syndrome coronavirus – Saudi Arabia. Geneva: (2015)

[31] Mackenzie JS Drury P Arthur RR Ryan MJ Grein T Slattery R . The global outbreak alert and response network. Glob Public Health. (2014) 9:1023–39. doi: 10.1080/17441692.2014.951870, 25186571 PMC4205922

[32] Ikejezie J Miglietta A Nezu IH Adele S Higdon MM Feikin D . Informing the pandemic response: the role of the WHO’S COVID-19 weekly epidemiological update. BMJ Glob Health. (2024) 9:4466. doi: 10.1136/bmjgh-2023-014466, 38580376 PMC11002403

[33] World Health Organization (WHO) In: WHO, editor. Disease outbreak news | Mpox - African region. Geneva: (2024)

[34] World Health Organization (WHO) In: WHO, editor. Disease outbreak news | Sudan virus disease - Uganda. Geneva: (2025)

[35] World Health Organization (WHO). International health regulations (2005). Third ed. Geneva: WHO (2016). Available: https://iris.who.int/bitstream/handle/10665/246107/9789241580496-eng.pdf?sequence=1 (Accessed August 11, 2025)

[36] World Health Organization (WHO). Strengthening surveillance of and response to foodborne diseases: a practical manual. stage 1: investigating foodborne disease outbreaks. Geneva: WHO (2017).

[37] Williams GS Impouma B Mboussou F Lee TMH Ogundiran O Okot C . Implementing epidemic intelligence in the WHO African region for early detection and response to acute public health events. Epidemiol Infect. (2021) 149:e261. doi: 10.1017/S095026882100114X, 33985609 PMC8727712

[38] Branda F Scarpa F Petrosillo N Ciccozzi M. A one health platform for future epidemic preparedness. Infectious Disease Reports. (2024) 16:281–8. doi: 10.3390/idr16020023, 38525770 PMC10961740

[39] World Health Organization (WHO) In: WHO, editor. Framework for a public health emergency operations Centre. Geneva: (2015)

[40] World Health Organization (WHO). Emergency response framework – 2nd Ed. Geneva: WHO (2017).

[41] Angulo FJ Cassell CH Tappero JW Bunnell RE. Progress and opportunities for strengthening global health security. Emerg Infect Dis. (2017) 23:758. doi: 10.3201/eid2313.171758 29155656 PMC5711315

[42] WHO African Region In: WHO African Region, editor. Technical guidelines for integrated disease surveillance and response in the WHO African region, booklet two: Sections 1, 2, and 3. Brazzaville: (2019)

[43] Cordes KM Cookson ST Boyd AT Hardy C Malik MR Mala P . Real-time surveillance in emergencies using the early warning alert and response network. Emerg Infect Dis. (2017) 23:S131–7. doi: 10.3201/eid2313.170446, 29155660 PMC5711309

[44] Wernli D Haustein T Conly J Carmeli Y Kickbusch I Harbarth S. A call for action: the application of the international health regulations to the global threat of antimicrobial resistance. PLoS Med. (2011) 8:e1001022. doi: 10.1371/journal.pmed.1001022, 21526227 PMC3079636

[45] World Health Organization (WHO) In: WHO, editor. WHO bacterial priority pathogens list, 2024: Bacterial pathogens of public health importance to guide research, development and strategies to prevent and control antimicrobial resistance. Geneva: (2024)

[46] Struelens MJ. The epidemiology of antimicrobial resistance in hospital acquired infections: problems and possible solutions. BMJ. (1998) 317:652 9727997 10.1136/bmj.317.7159.652 PMC1113836

[47] Sjolund-Karlsson M Reimer A Folster JP Walker M Dahourou GA Batra DG . Drug-resistance mechanisms in *Vibrio cholerae* O1 outbreak strain, Haiti, 2010. (special section: cholera in Haiti.). Emerg Infect Dis. (2011) 17:2151–4. doi: 10.3201/eid1711.110720, 22099122 PMC3310571

[48] Dadgostar P. Antimicrobial resistance: implications and costs. Infection and Drug Resistance. (2019) 12:3903–10. doi: 10.2147/IDR.S234610, 31908502 PMC6929930

[49] Wangai FK Masika MM Maritim MC Seaton RA. Methicillin-resistant *Staphylococcus aureus* (MRSA) in East Africa: red alert or red herring? BMC Infect Dis. (2019) 19:596. doi: 10.1186/s12879-019-4245-3, 31288757 PMC6617662

[50] Fukushige M Syue L-S Morikawa K Lin W-L Lee N-Y Chen P-L . Trend in healthcare-associated infections due to vancomycin-resistant Enterococcus at a Hospital in the era of COVID-19: more than hand hygiene is needed. J Microbiol Immunol Infect. (2022) 55:1211–8. doi: 10.1016/j.jmii.2022.08.003, 35989164 PMC9357275

[51] Fassbind DA Bioni GB Motta F Dias CAG. Clinical, microbiological characteristics and outcomes of Carbapenemase-producing Enterobacterales bloodstream infections in a pediatric Hospital in Brazil. BMC Infect Dis. (2025) 25:1253. doi: 10.1186/s12879-025-11696-7, 41063001 PMC12505604

[52] Ssekatawa K Byarugaba DK Wampande E Ejobi F. A systematic review: the current status of Carbapenem resistance in East Africa. BMC Res Notes. (2018) 11:629. doi: 10.1186/s13104-018-3738-2, 30170613 PMC6119249

[53] Hosaka Y Hirabayashi A Clark A Baker M Sugai M Stelling J . Enhanced automated detection of outbreaks of a rare antimicrobial-resistant bacterial species. PLoS One. (2024) 19:e0312477. doi: 10.1371/journal.pone.0312477, 39446801 PMC11500894

[54] Dortet L Agathine A Naas T Cuzon G Poirel L Nordmann P. Evaluation of the RAPIDEC® CARBA NP, the rapid CARB screen® and the Carba NP test for biochemical detection of Carbapenemase-producing Enterobacteriaceae. J Antimicrob Chemother. (2015) 70:3014–22. doi: 10.1093/jac/dkv213, 26260131

[55] Ellington MJ Ekelund O Aarestrup FM Canton R Doumith M Giske C . The role of whole genome sequencing in antimicrobial susceptibility testing of bacteria: report from the EUCAST subcommittee. Clin Microbiol Infect. (2017) 23:2–22. doi: 10.1016/j.cmi.2016.11.012, 27890457

[56] Iskandar K Molinier L Hallit S Sartelli M Hardcastle TC Haque M . Surveillance of antimicrobial resistance in low- and middle-income countries: a scattered picture. Antimicrob Resist Infect Control. (2021) 10:931. doi: 10.1186/s13756-021-00931-w, 33789754 PMC8011122

[57] Büchler AC Haddad Galas M Buetti N Alp E Apisarnthanarak A Dziekan G . Challenges and success stories of the implementation of infection control and antimicrobial stewardship strategies: proceedings of the 5th global ministerial summit on patient safety, 2023. Antimicrob Resist Infect Control. (2024) 13:16. doi: 10.1186/s13756-023-01344-7, 38331974 PMC10854024

[58] Kasujja H Waswa JP Kiggundu R Murungi M Kwikiriza G Bahatungire R . Enhancing infection prevention and control through hand hygiene compliance in six Ugandan hospitals using quality improvement approaches. Front Public Health. (2024) 12:1465439. doi: 10.3389/fpubh.2024.1465439, 39502813 PMC11534609

[59] World Health Organization (WHO). Carbapenem-Resistant *Pseudomonas Aeruginosa* Infection - Mexico. Geneva: WHO (2019).

[60] Wilder-Smith A Osman S. Public health emergencies of international concern: a historic overview. J Travel Med. (2020) 27:227. doi: 10.1093/JTM/TAAA227, 33284964 PMC7798963

[61] Royal College of Pathologists. Antimicrobial resistance: The slow pandemic. London: Royal College of Pathologists (2023).

[62] The Week Staff. The rise of the superbugs: Why antibiotic resistance is a ‘slow-moving pandemic. London: The Week (2021).

[63] Moyo E Mhango M Moyo P Dzinamarira T Chitungo I Murewanhema G. Emerging infectious disease outbreaks in sub-Saharan Africa: learning from the past and present to be better prepared for future outbreaks. Front Public Health. (2023) 11:986. doi: 10.3389/fpubh.2023.1049986, 37228735 PMC10203177

[64] Garg SK. Antibiotic misuse during COVID-19 pandemic: a recipe for disaster. Indian J Critical Care Med. (2021) 25:617–9. doi: 10.5005/jp-journals-10071-23862, 34316138 PMC8286398

[65] Nandi A Pecetta S Bloom DE. Global antibiotic use during the COVID-19 pandemic: analysis of pharmaceutical sales data from 71 countries, 2020–2022. eClinicalMedicine. (2023) 57:848. doi: 10.1016/j.eclinm.2023.101848, 36776504 PMC9900305

[66] Massarine NCM de Almeida Souza GH Nunes IB Salomé TM dos Santos Barbosa M Faccin I . How did COVID-19 impact the antimicrobial consumption and bacterial resistance profiles in Brazil? Antibiotics. (2023) 12:374. doi: 10.3390/antibiotics12091374 37760671 PMC10526034

[67] Pandak N Al Sidairi H Al-Zakwani I Al Balushi Z Chhetri S Ba’Omar M . The outcome of antibiotic overuse before and during the COVID-19 pandemic in a tertiary care hospital in Oman. Antibiotics. (2023) 12:665. doi: 10.3390/antibiotics12121665, 38136699 PMC10740960

[68] Magnano San Lio R Favara G Maugeri A Barchitta M Agodi A. How antimicrobial resistance is linked to climate change: an overview of two intertwined global challenges. Int J Environ Res Public Health. (2023) 20:681. doi: 10.3390/ijerph20031681, 36767043 PMC9914631

[69] Millar M. Inequality and antibiotic resistance: a Contractualist perspective. Bioethics. (2019) 33:749–55. doi: 10.1111/bioe.12654, 31423607

[70] Topluoglu S Taylan-Ozkan A Alp E. Impact of wars and natural disasters on emerging and re-emerging infectious diseases. Front Public Health. (2023) 11:929. doi: 10.3389/fpubh.2023.1215929, 37727613 PMC10505936

[71] Yusuff SI Tajudeen YA Oladunjoye IO Oladipo HJ Bolarinwa OV Popoola OT . The need to increase antimicrobial resistance surveillance among forcibly displaced persons (FDPs). Tropical Dis Travel Med Vaccines. (2023) 9:12. doi: 10.1186/s40794-023-00198-6, 37653439 PMC10472691

[72] Neila G Muhvich Johnathan Ching Carly Gomez Bridget Horvath Evan Nahum Yanina . Effects of microplastic concentration, composition, and size on *Escherichia coli* biofilm-associated antimicrobial resistance. Appl Environ Microbiol. (2025) 1:e02282–24. doi: 10.1128/aem.02282-24 PMC12016508 40067049

[73] Church DL. Major factors affecting the emergence and re-emergence of infectious diseases. Clin Lab Med. (2004) 24:559–86. doi: 10.1016/j.cll.2004.05.008, 15325056 PMC7119055

[74] Wailan AM Paterson DL. The spread and acquisition of NDM-1: a multifactorial problem. Expert Rev Anti-Infect Ther. (2014) 12:91–115. doi: 10.1586/14787210.2014.856756, 24308710

[75] Wang Y Tian GB Zhang R Shen Y Tyrrell JM Huang X . Prevalence, risk factors, outcomes, and molecular epidemiology of Mcr-1-positive Enterobacteriaceae in patients and healthy adults from China: an epidemiological and clinical study. Lancet Infect Dis. (2017) 17:390–9. doi: 10.1016/S1473-3099(16)30527-8, 28139431

[76] World Health Organization (WHO). Antimicrobial Resistance, Hypervirulent *Klebsiella Pneumoniae* - Global Situation. Geneva: WHO (2024).

[77] Obenhuber T Scheier TC Stutz T Hug M Fontein D Kaiser A . An outbreak of multi-drug-resistant *Acinetobacter Baumannii* on a burns ICU and its control with multi-faceted containment measures. J Hosp Infect. (2024) 146:102–8. doi: 10.1016/j.jhin.2024.01.002, 38219836

[78] Centers for Disease Control and Prevention (CDC). CDC health alert network (HAN) health advisory: Increase in extensively drug-resistant Shigella infections (shigellosis) in the United States. Atlanta, GA: CDC (2023).

[79] The Conversation. (2025). “There’s an outbreak of Melioidosis in North Queensland. Here’s what to know about this deadly ‘mud bug.’” Available online at: https://theconversation.com/theres-an-outbreak-of-melioidosis-in-north-queensland-heres-what-to-know-about-this-deadly-mud-bug-250392 (acessed March 31, 2025).

[80] Sivabalan P Satyaputra F Gassiep I Forde B Frazer J Glover M . Meropenem-resistant *Burkholderia pseudomallei*: a concerning single case in Australia with no prior meropenem exposure. Access Microbiol. (2024) 6:619. doi: 10.1099/acmi.0.000619.v4 PMC11652719 39698509

[81] Pan American Health Organization (PAHO). Epidemiological alert: Update on the cholera situation in Haiti and Dominican Republic. Washington, D.C.: PAHO (2011).

[82] World Health Organization (WHO). Global antimicrobial resistance and use surveillance system (GLASS) report 2022. Geneva: WHO (2022).

[83] World Health Organization (WHO). Comprehensive review of the WHO global action plan on antimicrobial resistance: evaluation brief – September 2021. Geneva: WHO (2021).

[84] Kayiwa J Homsy J Nelson LJ Ocom F Kasule JN Wetaka MM . Establishing a public health emergency operations Center in an Outbreak-Prone Country: lessons learned in Uganda, January 2014 to December 2021. Health Security. (2022) 20:394–407. doi: 10.1089/hs.2022.0048, 35984936 PMC10985018

